# Association of Serum Level of Growth Differentiation Factor 15 with Liver Cirrhosis and Hepatocellular Carcinoma

**DOI:** 10.1371/journal.pone.0127518

**Published:** 2015-05-21

**Authors:** Xiuying Liu, Xiumei Chi, Qiaoling Gong, Lei Gao, Yuqiang Niu, Xiaojing Chi, Min Cheng, Youhui Si, Maorong Wang, Jin Zhong, Junqi Niu, Wei Yang

**Affiliations:** 1 MOH Key Laboratory of Systems Biology of Pathogens, Institute of Pathogen Biology, Chinese Academy of Medical Sciences & Peking Union Medical College, Beijing, China; 2 First Hospital, Jilin University, Changchun, China; 3 Liver Disease Center of PLA, the 81st Hospital of PLA, Nanjing, China; 4 Institut Pasteur of Shanghai, Chinese Academy of Sciences, Shanghai, China; University Medical Center of Princeton/Rutgers Robert Wood Johnson Medical School, UNITED STATES

## Abstract

Hepatocellular carcinoma (HCC) and liver cirrhosis are associated with high mortality worldwide. Currently, alpha-fetoprotein (AFP) is used as a standard serum marker for the detection of HCC, but its sensitivity and specificity are unsatisfactory, and optimal diagnostic markers for cirrhosis are lacking. We previously reported that growth differentiation factor 15 (GDF15) was significantly induced in HCV-infected hepatocytes. This study aimed to investigate GDF15 expression and its correlation with hepatitis virus-related liver diseases. A total of 412 patients with various liver diseases were studied. Healthy and *Mycobacterium tuberculosis*-infected subjects were included as controls. Serum and tissue GDF15 levels were measured. Serum GDF15 levels were significantly increased in patients with HCC (6.66±0.67 ng/mL, *p*<0.0001) and cirrhosis (6.51±1.47 ng/mL, *p*<0.0001) compared with healthy controls (0.31±0.01 ng/mL), though the GDF15 levels in HBV and HCV carriers were moderately elevated (1.34±0.19 ng/mL and 2.13±0.53 ng/mL, respectively). Compared with HBV or HCV carriers, GDF15 had a sensitivity of 63.1% and a specificity of 86.6% at the optimal cut-off point of 2.463 ng/mL in patients with liver cirrhosis or HCC. In HCC patients, the area under the receiver operating curve was 0.84 for GDF15 and 0.76 for AFP, but 0.91 for the combined GDF15 and AFP. Serum GDF15 levels did not significantly differ between the high-AFP and low-AFP groups. GDF15 protein expression in HCC was significantly higher than that in the corresponding adjacent paracarcinomatous tissue and normal liver. Using a combination of GDF15 and AFP will improve the sensitivity and specificity of HCC diagnosis. Further research and the clinical implementation of serum GDF15 measurement as a biomarker for HCC and cirrhosis are recommended.

## Introduction

Hepatitis B virus (HBV) and hepatitis C virus (HCV) infections are major risk factors for liver cirrhosis (LC) and hepatocellular carcinoma (HCC), which are the most common causes of end-stage liver disease. The survival rate of HCC is 12–18% in USA depending on race, placing it among the cancers with the worst prognosis.[1] However, the prognosis can be significantly improved by early diagnosis and treatment. Patients suspected of having LC or HCC typically undergo clinical examinations, including abdominal CT and liver biopsy. CT can detect lesion growth, but it is only a surrogate marker and may delay addressing the diagnosis.[2] Liver biopsy is an accurate diagnostic method, but its associated disadvantages, including invasion and poor patient compliance, limit its broad application.[3] Therefore, the characterization of novel non-invasive biomarkers for LC and HCC is urgently needed.

Screening early serological markers in patients at high risk for developing LC or HCC may decrease cancer-related mortality and reduce medical costs. To date, only a few serum biomarkers are available for the diagnosis of HCC, including the oncofetal glycoprotein alpha-fetoprotein (AFP). However, the sensitivity and specificity of AFP are not sufficient to meet the requirements of a perfect tumor biomarker. [4, 5] Using a genome-wide gene expression profiling method, our previous study demonstrated that growth differentiation factor 15 (GDF15), a divergent member of the transforming growth factor β (TGF-β) superfamily, was significantly induced in HCV-infected hepatocytes *in vitro*.[6]

GDF15, also known as macrophage inhibitory cytokine-1 or nonsteroidal anti-inflammatory drug-activated gene, was first isolated in macrophages based on its increased mRNA expression after cytokine stimulation.[7] Endogenous expression of GDF15 is regulated by several tumor-related pathways, including p53/DEC1, GSK-3β and EGR-1.[8–12] Similar to other TGF-β superfamily cytokines, GDF15 is synthesized as a precursor protein. Proper proteolysis produces the N-terminal propeptide from the mature GDF15 peptide, which is then secreted as a disulfide-linked homodimer.[7, 13] The membrane receptor of GDF15 has not been identified, but activation of intracellular Akt is considered as a major GDF15-stimulated pathway.[14] Importantly, other groups have also suggested that the serum GDF15 level can be used as a risk factor for some cancers, including prostate, colorectal, pancreatic, lung and breast cancer as well as melanoma,[15–24] and other physiological and pathological processes, including heart failure, diabetes, body weight regulation and aging.[25–29] However, the potential value of GDF15 as a serum biomarker in hepatitis virus-related LC and HCC has not been described.

In the present study, a large multicenter evaluation was conducted to compare the serum GDF15 levels in healthy subjects and patients with hepatitis virus-related liver diseases. Immunoassays were used to determine the origin of GDF15 in HCC tissues and their corresponding paracarcinomatous tissues.

## Materials and Methods

### Study population

In this multicenter retrospective study, healthy subjects and patients were recruited from Beijing (North China), Jilin (Northeast China), Jiangsu (East China) and Guangxi (Southwest China) provinces. A total 1014 subjects were recruited comprising healthy controls (n = 202); patients with HCC (n = 223), including HBV-positive (n = 164), HCV-positive (n = 30) and HBV/HCV-negative (n = 29) patients; patients with LC (n = 88); HBV carriers (n = 51); HCV carriers (n = 50); and patients with active tuberculosis (TB, n = 200) or latent TB infection (n = 200). HCC and LC were diagnosed based on the guidelines of the Chinese Society of Hepatology and the Chinese Society of Infectious Diseases, Chinese Medical Association. All of the HCC and LC patients were confirmed with CT or magnetic resonance imaging or following biopsy results. Grading of HCC was based on the tumor differentiation according to Edmondson and Steiner system. All of the healthy volunteers were tested negative for HBV and HCV nucleic acids. Molecular diagnoses of HBV and HCV carriers were made by measuring serum viral nucleic acids. The study protocol was reviewed and approved by the Ethics Committees of the Institute of Pathogen Biology Chinese Academy of Medical Sciences, the first Hospital of Jilin University and the 81st Hospital of PLA. Written informed consent was obtained from the subjects. The age (mean ± SD) and gender (male: female) of each group were summarized in Table 1.

**Table 1:** Major characteristics of the study population.

Variables	Healthy controls	HBV-Hepatocirrhosis cases	HBV-/HCV- HCC cases	HBV+/HCV- HCC cases	HBV-/HCV+ HCC cases	p for difference
Sample size	183	60	27	164	27	
Mean age (SD)	50 (15.0)	50.3 (9.0)	56.5 (11.5)	50.8 (10.0)	65.6 (10.7)	p<0.0001 (ANOVA)
Frequency of males (%)	92 (50.3)	42 (70.0)	21 (77.8)	145 (88.4)	19 (70.4)	p<0.0001 (chi-square test)

### Samples

To detect serum GDF15, blood samples were obtained from hospitals, and serum was stored at -80°C until use. All of the blood samples were collected in accordance with the approval of the Ethics Committee at each institution. Detailed physiological and diagnostic information was recorded for all patients and controls. The control group consisted of normal healthy subjects with no known history of cancer or HBV or HCV infection. For tissue GDF15 immunohistochemistry, 20 HCC tissue samples of tumor grades 1–3 and 10 normal liver (NL) tissue samples were used. For Western blotting analysis of GDF15, 6 pairs of HBV-positive and 6 pairs of HCV-positive HCC tissue samples and adjacent paracarcinomatous liver (PCL) tissue samples were obtained from the First Hospital of Jilin University. All HCC samples were pathologically confirmed.

### Enzyme-linked immunosorbent assay (ELISA)

GDF15 was measured in serum using a specific ELISA kit (Cat# DY957, R&D Systems, Minneapolis, MN), according to the manufacturer’s recommended protocol. Briefly, standard curves were produced from standards provided with the kit and serially 2-fold diluted from 500 pg/mL to 2 pg/mL. The minimal detection limit of this assay was 2 pg/mL, and the linear range of detection is 2–500 pg/mL. Samples with readings greater than that of the highest standard were diluted appropriately so that the values were within the standard curve. 100 μl various concentrations of standard recombinant human GDF15 and serum samples were added to each well of a 96-well plate that has been coated with 2 μg/mL of Capture Antibody for 2 h at 37°C, After the plate was washed, 50 ng/mL Detection Antibody was added and incubated for another 2 h at 37°C. Finally, Streptavidin-HRP was added and the optical density was read at 450 nm with an absorbance correction at 540 nm in multifunctional microplate reader SpectraMax M5 (Molecular Devices, Sunnyvale, CA). Clinical data were blinded to the laboratory, and each sample was tested in duplicate. All the tests were done at a central site for better quality control.

### Immunohistochemistry (IHC)

Tumor tissue blocks were fixed in 3% paraformaldehyde and embedded in paraffin. Normal tissue was obtained from the normal livers of adults who died suddenly. To reduce selection bias, two independent histopathologists reviewed areas of malignant tissues, adjacent normal tissues and normal liver tissue. Tissue sections (5 μm thick) were obtained from paraffin-embedded tissue blocks, baked in a 60°C thermostat for 30 minutes, dewaxed and hydrated using conventional methods. Antigen retrieval was performed using the heat-induced epitope retrieval method in 0.01 M citrate buffer (pH = 6.0). The sections were then incubated with 3% H_2_O_2_ at room temperature for 10 minutes. Rabbit antibody against GDF15 (1:50 dilution, Cat# HPA011191, Sigma-Aldrich Co.; St. Louis, MO) or normal rabbit IgG isotype and HRP-conjugated goat anti-rabbit IgG were used. Slides were developed with diaminobenzidine, counterstained in hematoxylin, dehydrated and mounted in Neo-Mount.

### Western blotting

For the analysis of GDF15 expression in HCC tissues, clinical tissue specimens were taken from freshly isolated surgical resections and stored at -80°C until use. Frozen tissues were thawed and resuspended in RIPA buffer (25 mM Tris•HCl pH 7.6, 150 mM NaCl, 1% NP-40, 1% sodium deoxycholate, 0.1% sodium dodecyl sulfate) with protease inhibitors (Roche Applied Science, Indianapolis, IN, USA). After homogenization on ice and protein quantification, tissue lysates containing 30 μg of protein were used for Western blotting as described previously.[30] The primary anti-human GDF15 polyclonal antibody was purchased from Sigma-Aldrich.

### Statistical analysis

GDF15 concentrations were compared using Student’s *t-*test. The data are presented as the mean ± SD. The mean ages of the studied groups were examined for differences by analysis of variance (ANOVA). The Chi-squared test was used to assess differences in the frequency of males among groups. Differences between age- and sex-adjusted mean values of serum GDF15 levels according to potential predictors or clinical characteristics of HCC (serum level of AFP, tumor size, viral load, smoking, alcohol consumption, family history of HCC and history of IFN treatment) were examined by analysis of covariance, and trends were assessed by linear regression analysis. To compare the diagnostic values of GDF15, receiver operating characteristic (ROC) curves were generated, the areas under the curves were calculated, and clinically relevant threshold levels were chosen to optimize sensitivity and specificity. All statistical analyses were carried out using SAS statistical software (SAS Institute Inc, Cary, NC), release 9.1, and GraphPad Prism, version 5.01 for Windows (GraphPad Software; San Diego, California).

## Results

### The serum GDF15 level is elevated in patients with LC or HCC

**Fig 1:**
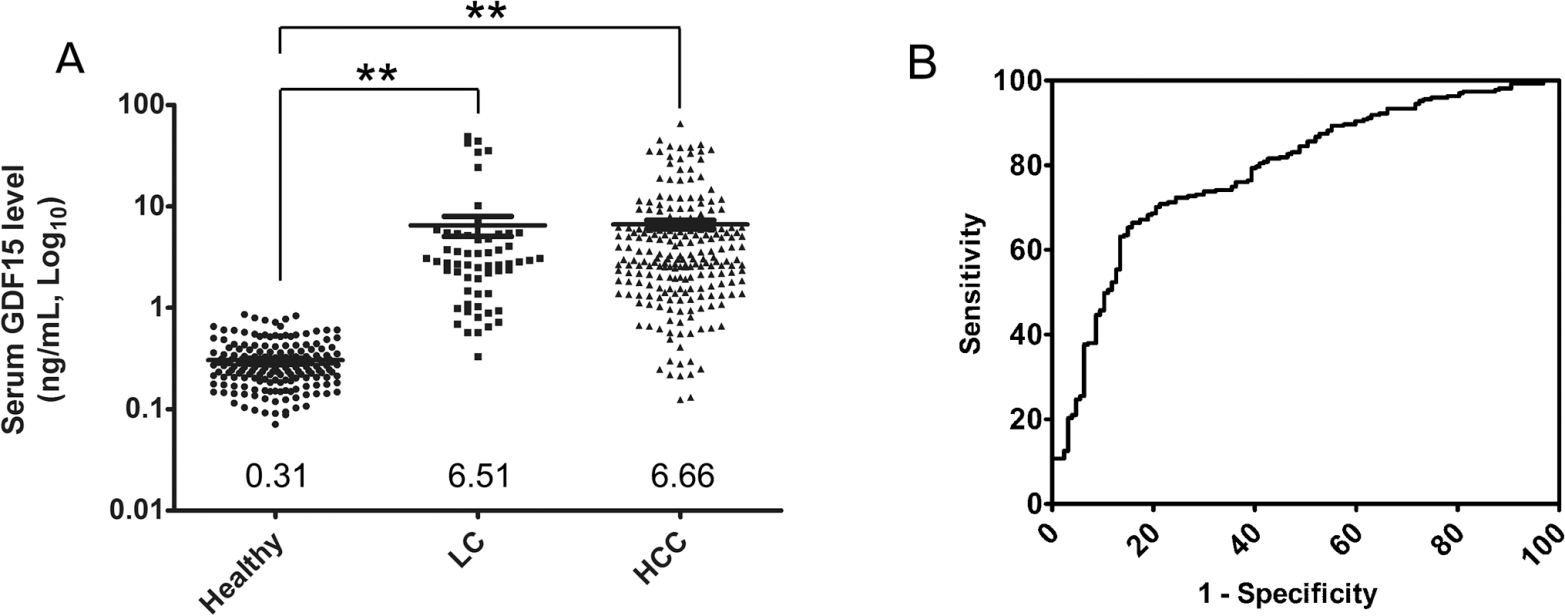
Serum GDF15 is increased in patients with LC or HCC. **(A)** Scatter plots show the serum GDF15 levels in healthy (n = 202), LC (n = 88) and HCC (n = 223) patients by ELISA. The black line indicates the mean, for which the value is indicated at the bottom of the scatter plot. ** indicates that *P* < 0.0001 for both LC and HCC compared with healthy controls. **(B)** The GDF15 ROC curve is plotted to compare the HCC or LC patients versus HBV or HCV carriers.

Our previous findings suggested an up-regulation of GDF15 in an HCV-infected cell culture model and a role of GDF15 in regulating HCC-related genes.[6] Accumulating evidence indicates that GDF15 as a risk factor is up-regulated in multiple diseases. However, a clinical relevance has not been reported between serum GDF15 level and severe liver diseases, such as hepatocellular carcinoma and cirrhosis. In this study, to investigate the potentially indicative role of GDF15 in severe liver diseases, 202 healthy subjects, 223 patients with HCC and 88 patients with LC were recruited between 2010–2011. The comparison of basic demographics of the study groups was summarized in Table 1. The mean±SEM of serum GDF15 in the healthy subjects was 0.31±0.01 ng/mL (Fig 1A). In contrast, patients with LC and HCC had 6.51±1.47 ng/mL (*p*<0.0001) and 6.66±0.67 ng/mL (*p*<0.0001) GDF15, respectively, which was significantly higher than that of healthy subjects (Fig 1A). In healthy subjects, GDF15 was not correlated with age or gender (data not shown).

To determine the sensitivity and specificity of serum GDF15 for categorizing patients with HCC or LC versus HBV and HCV carriers, an ROC curve was plotted to define the optimal cut-off. The area under the ROC curve (AUROC) was 0.7882 (95% CI 0.7409 to 0.8354), with a sensitivity of 63.1% (95% CI 57.05% to 68.86%), specificity of 86.61% (95% CI 79.43% to 92.00%), and an optimal cut-off value of 2.463 ng/mL (Fig 1B). The serum GDF15 level effectively differentiated patients with LC or HCC from patients in the cohort with chronic HBV or HCV carriers.

### The serum GDF15 level is associated with hepatitis virus-related liver diseases

**Fig 2:**
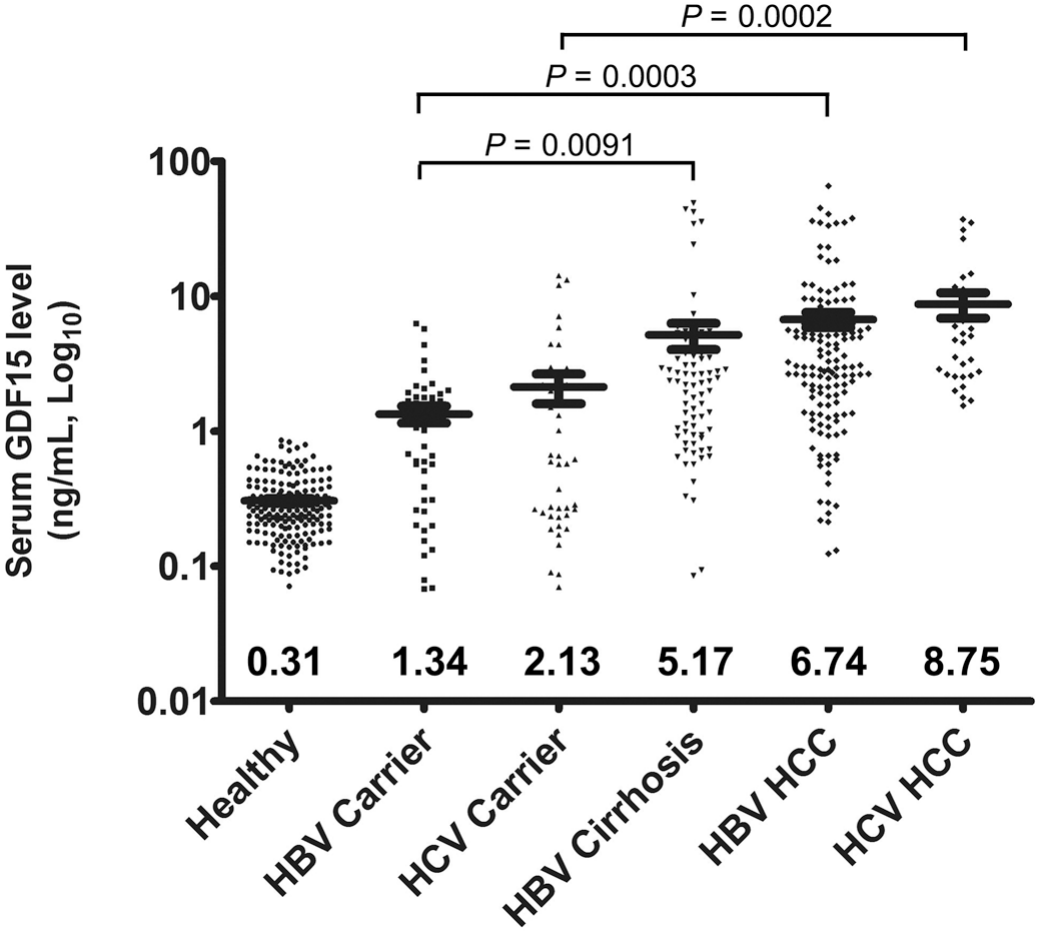
Serum GDF15 level alterations in patients with hepatitis virus-related diseases. Scatter plots show the serum GDF15 levels in healthy subjects and patients with various liver diseases by ELISA. The black line indicates the mean, for which the value is indicated at the bottom of the scatter plot. The *P* values for HBV carriers vs. HBV cirrhosis, HBV carriers vs. HBV HCC and HCV carriers vs. HCV HCC are indicated.

**Fig 3:**
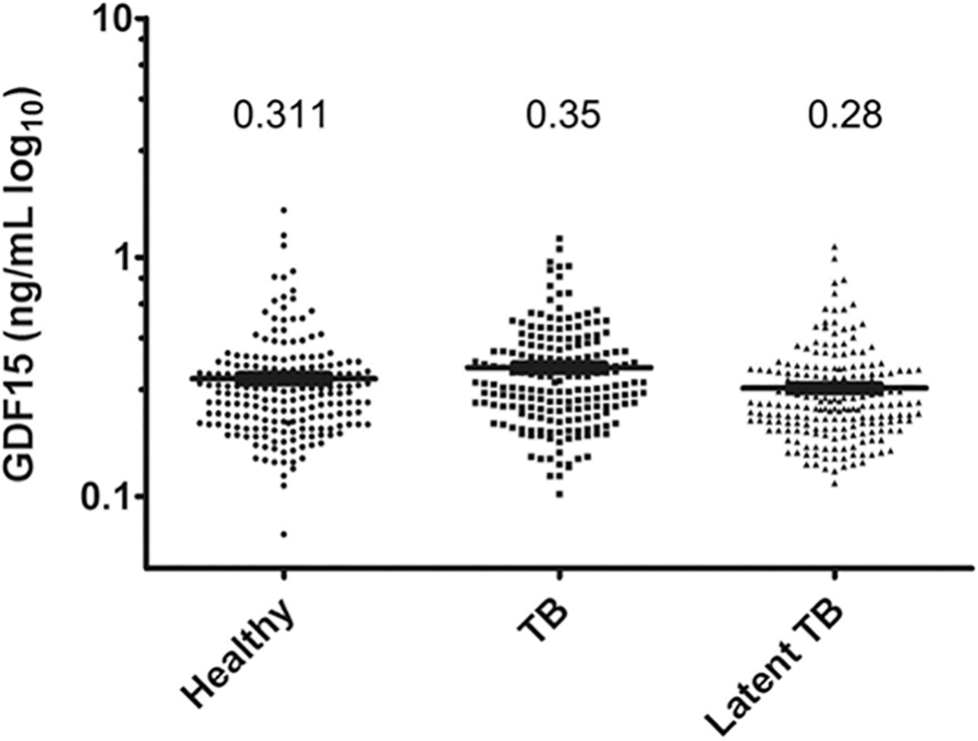
Serum GDF15 is not related to tuberculosis infection. Scatter plots showing the serum GDF15 levels in healthy subjects (n = 202) and patients with active tuberculosis (TB, n = 200) or latent TB (n = 200) infection by ELISA. The black line indicates the mean, for which the value is indicated on the top of the scatter plot.

A large proportion of HCC and cirrhosis cases are associated with chronic hepatitis virus infection, particularly HBV and HCV. To further investigate the relationship of GDF15 expression and hepatitis virus-related liver diseases, serum GDF15 values were measured in HBV carriers, HCV carriers and patients with HBV cirrhosis, HBV HCC, HCV cirrhosis or HCV HCC. As shown in Fig 2, moderate elevations of serum GDF15 were observed in HBV carriers and HCV carriers, with values of 1.34±0.19 ng/mL and 2.13±0.53 ng/mL (*p* = 0.1463), respectively. Notably, with the exception of HBV cirrhosis and HBV HCC, HBV HCC and HCV HCC and HBV cirrhosis and HCV HCC, the disease groups significantly differed from each other (p<0.001). In this study, due to the limitation of the small number of available HCV cirrhosis samples, the data of HCV cirrhosis was omitted in Fig 2. The highest GDF15 level was found in the HCV-positive HCC group. Chronic infection by microbes, such as hepatitis viruses and TB, often correlates with inflammation and immune cell dysfunction. To investigate the relationship between elevated GDF15 in hepatitis virus-related diseases and carcinogenesis or microbial infection, we analyzed the serum GDF15 levels in patients with active or latent TB infection. No statistically significant difference was observed between the healthy and TB groups, with GDF15 values of 0.31±0.02 ng/mL in healthy subjects, 0.35±0.01 ng/mL (*p* = 0.0532) in active TB patients and 0.28±0.01 ng/mL (*p* = 0.1089) in latent TB patients (Fig 3).

### Combined use of AFP and GDF15 increases the diagnostic sensitivity and specificity for HCC

**Fig 4:**
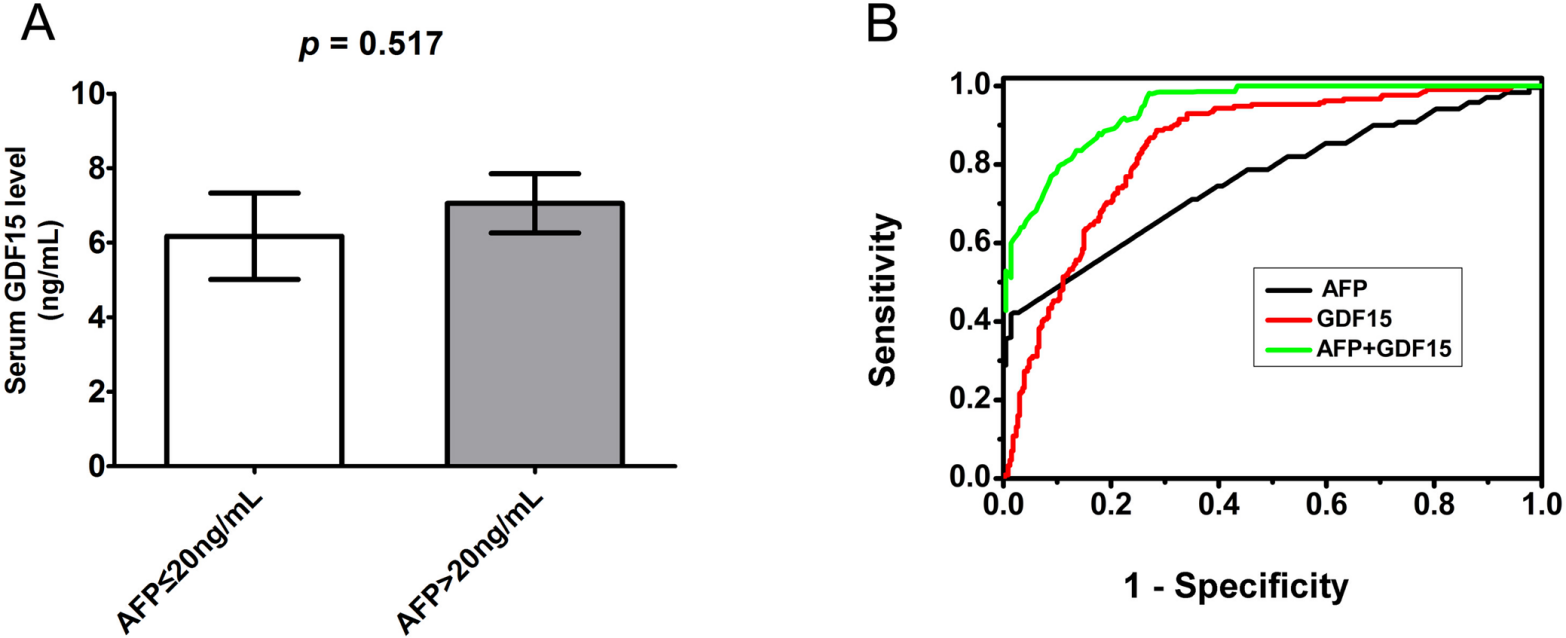
Comparison of serum GDF15 and AFP for the diagnosis of HCC. **(A)** Serum GDF15 levels in two HCC groups with AFP ≤20 ng/mL (n = 91) and >20 ng/mL (n = 132), respectively. **(B)** ROC curves comparing GDF15 (red line), AFP (black line) and combined (green line) for HCC diagnosis versus all other conditions in the cohort, including healthy patients, HBV or HCV carriers and patients with cirrhosis. The area under the ROC curve for GDF15 was significantly different from that for AFP, *P*<0.001.

For clinically diagnosing HCC, the standard serum screening test for AFP has poor sensitivity for the detection of small tumors, and AFP is often elevated in patients with chronic HCV infection in the absence of HCC.[31] We therefore evaluated the ability of the combination of GDF15 and AFP to improve HCC diagnosis. In the HCC group, 132 of 223 (59.19%) patients had an AFP level >20 ng/mL. We therefore divided the HCC group using a cut-off of 20 ng/mL AFP and analyzed the serum GDF15 level in both groups. As shown in Fig 4A, the low and high AFP groups (≤20 and >20 ng/mL, respectively) had serum GDF15 levels of 6.17±1.16 ng/mL and 7.05±0.79 ng/mL, respectively, with no significant difference (*p* = 0.517) (Fig 4A). We then constructed ROC curves for AFP, GDF15 and combination in HCC patients and compared them with all of the other conditions in this cohort. The AUROC for AFP was 0.7606 (95% CI 0.7172 to 0.8041), and the AUROC for GDF15 was 0.8426 (95% CI 0.8091 to 0.8761), with a sensitivity of 86.79% (95% CI 81.48% to 91.04%), a specificity of 72.75% (95% CI 67.64% to 77.46%) and an optimal cut-off value of 1.945 ng/mL (Fig 4B). Interestingly, combination use of AFP and GDF15 will greatly improve the diagnosis performance for HCC with AUROC 0.9101 (Fig 4B). These results suggest that the serum GDF15 level is a good alternative to AFP for the differentiation of HCC from other liver diseases. Additionally, using both AFP and GDF15 may improve the sensitivity and specificity of HCC diagnosis.

### Correlation between serum GDF15 expression and HCC features

We analyzed the possible correlation between the serum GDF15 level and the clinicopathologic or biological features of the HCC cases. The results are summarized in Table 2. Overall, we found no significant correlation between the serum GDF15 level and the selected parameters. However, trends toward relatively high mean serum GDF15 levels were observed in patients with a larger tumor size or higher viral load, current and former smokers and patients who consumed alcohol or had a family history of HCC (Table 2).

**Table 2:** Serum level of GDF15 among HBV/HCV related HCC cases.

Variables (n^a^)		Adjusted means (Std error) (ng/mL)	p for difference (p for linear trend)
Serum AFP Level			
	<20ng/mL (91)	6.17 (1.16)	0.517
	≥20ng/mL (132)	7.05 (0.79)	(0.168)
Tumor size			
	<3 cm (43)	4.64 (1.57)	0.526
	≥3–5 cm (29)	7.06 (1.92)	(0.078)
	≥5 cm or multiple tumor (65)	8.24 (1.27)	
Viral load^b^			
	<500 copies/mL (27)	4.72 (2.03)	0.789
	500–14500 copies/mL (34)	6.05 (1.85)	
	14500–605000 copies/mL (38)	8.45 (1.72)	(0.044)
	>605000 copies/mL (29)	7.10 (1.96)	
Smoke			
	Never smoker (134)	6.20 (0.88)	0.106
	Current or former smoker (46)	9.08 (1.52)	
Alcohol drinking			
	No (123)	6.75 (0.92)	0.670
	Yes (65)	7.43 (1.28)	
Family history of HCC			
	No (157)	6.60 (0.81)	0.215
	Yes (30)	9.14 (1.87)	
IFN treatment			
	No (164)	7.18 (0.80)	0.488
	Yes (6)	4.21 (4.20)	

^a^ Sum may not always add up to total because of missing values.

^b^Only HBV positive cases were included in this analysis.

### GDF15 protein expression in HCC, PCL and NL

**Fig 5:**
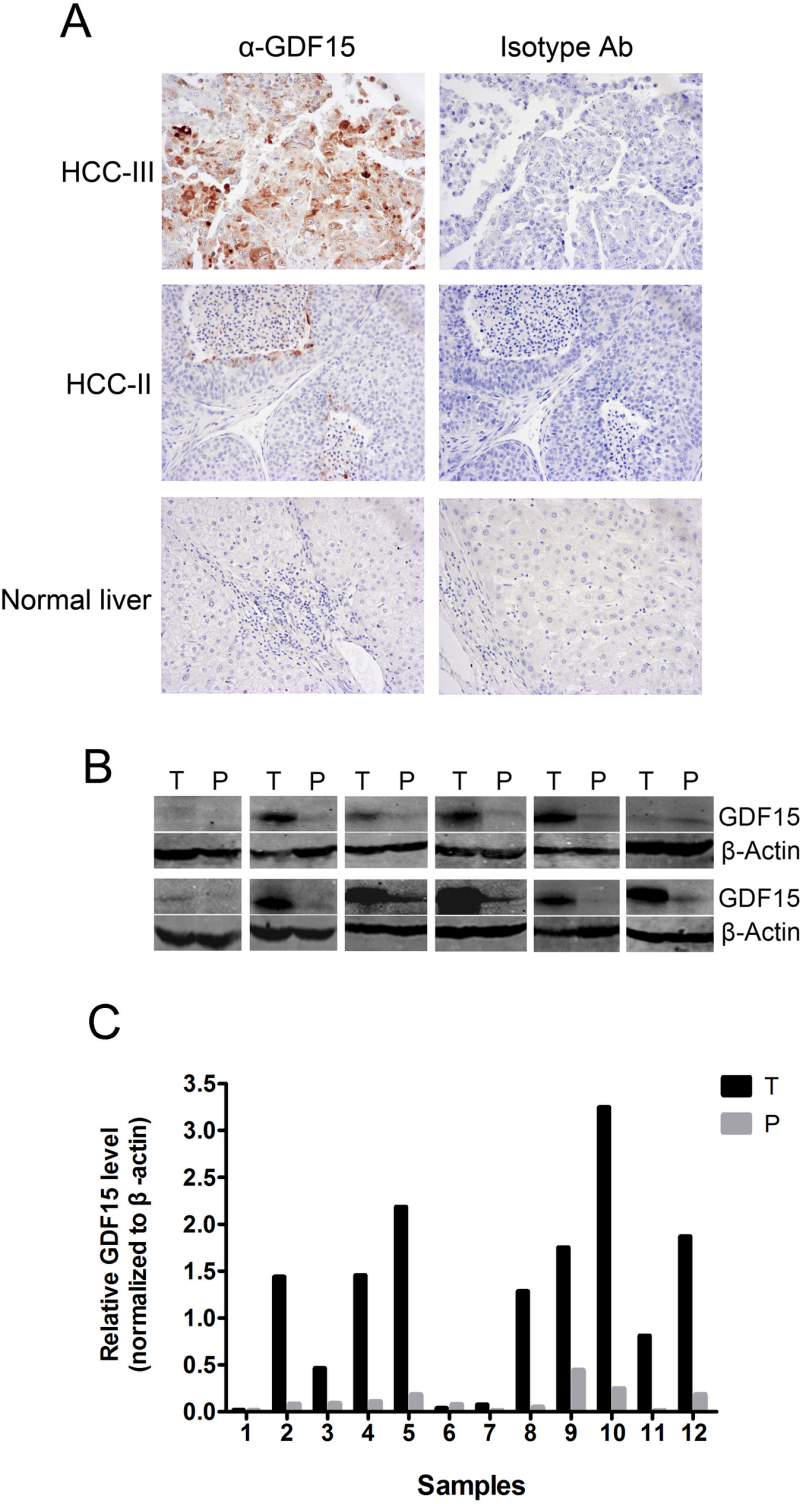
GDF15 protein expression in HCC, adjacent paracarcinomatous liver (PCL) and normal liver (NL) tissues. **(A)** Normal liver and HCC with different grades of malignancy were prepared for IHC with anti-GDF15 or isotype antibodies, respectively. Representative results are shown. **(B)** GDF15 protein expression in HCC (T) and PCL (P) lysates was determined by Western blotting. An antibody against β-Actin was used as a loading control. **(C)** Quantification of Western Blot described in Fig 4B with Image J.

In this study, we showed evidence that the serum level of GDF15 was significantly elevated in the patients with HCC and cirrhosis. However, the sources of this up-regulated GDF15 were still unclear. It may be produced from either malignant tissues or systematic stress. Therefore, to identify whether the expression of GDF15 was also increased in HCC, IHC and Western blotting were performed on normal and malignant tissues. Twenty HCC biopsy tissues, 10 HBV-positive and 10 HCV-positive, were used for IHC with an antibody against GDF15. Normal liver tissues were used as control. Grading of HCC was based on the tumor differentiation according to Edmondson and Steiner system. Basically, performance of the antibody for IHC was not satisfactory very well. Only 5 of 20 (25%) samples were positively stained at various intensities. The representative data were shown in Fig 5A. Expression of GDF15 was increased in relatively moderate (HCC-II) or severe (HCC-III) malignant HCCs, but not in the normal liver (Fig 5A). To further confirm the IHC data, Western blotting was performed, and 12 pairs of HCC (T) and PCL (P) were prepared from the same resection specimen to eliminate the effects of differences in the genetic background. GDF15 protein levels were consistently increased in 9 of 12 (75%) HCC samples (Fig 5B). In addition, we did quantification of GDF15 levels of Western blotting films by analyzing the band density with image J software and the results were shown in Fig 5C. These results suggest that the elevated serum GDF15 level may result from GDF15 production by HCC tissues.

## Discussion

Currently, HCC is the fifth most common cancer in the world, and decompensated cirrhosis often carries a high risk of death. There is an urgent need for improved tools for early and non-invasive diagnosis. In this study, we showed that GDF15, a divergent TGF-β superfamily member, was markedly increased in the serum of patients with HCC or LC. Among the patients with chronic HBV or HCV infection, serum GDF15 levels were also moderately higher than the healthy controls but significantly lower than those with HCC and cirrhosis. This *in vivo* finding confirms our previous observation that GDF15 was up-regulated in HCV-infected hepatocytes.[6] The ROC curve comparing patients with HCC or LC versus HBV or HCV carriers in the cohort demonstrated that GDF15 was able to discriminate HCC and LC with an AUROC of 0.788. These findings suggest that GDF15 may be a serum marker of HCC or LC. We also attempted to determine the potential value of GDF15 in HCC diagnosis. The ROC curve comparing HCC patients with patients with any other condition (including healthy controls, HBV or HCV carriers and patients with cirrhosis) showed that GDF15 was superior to AFP in diagnosing HCC in this cohort, with an AUROC of 0.84 (95% CI 0.8091 to 0.8761), a sensitivity of 86.79% and a specificity of 72.75%. More importantly, we found serum GDF15 levels above the optimal cut-off (1.945 ng/mL) in 81.3% and 85.9% of HCC patients with AFP levels below 20 ng/mL and 100 ng/mL, respectively, demonstrating the utility of GDF15 in the diagnosis of HCC in patients with normal or mildly elevated AFP. In addition, we found AFP and GDF15 combination indeed achieved improvement compared with the single factor analysis. This finding has value in screening HCC risk in early, large population, instead in the liver cirrhosis patients.

Previous studies have shown that the normal reference range for serum GDF15 in healthy subjects is approximately 0.2–1.2 ng/mL,[15] which is consistent with our results. As a risk factor, increased serum GDF15 has been associated with several human diseases, including cardiovascular diseases and prostate,[17, 25, 32, 33] breast and colon cancer.[18, 24] To our knowledge, this is the first report demonstrating an association between serum GDF15 levels and severe liver diseases, including HCC and LC. Our data showed increased GDF15 expression in both the serum and biopsy tissues of HCC patients. However, the detailed mechanism of GDF15 induction in HCC remains unclear. The overexpression of p53 has been associated with the pathogenesis of HCC and even with chronic HCV infection and LC. [34, 35] p53 can bind to the promoter region of GDF15 and powerfully induce GDF15 expression both *in vitro* and *in vivo*, [8, 36] which is consistent with the phenomenon of the GDF15 overexpression in HCC. Even so, the biological significance of GDF15 induction in HCC pathogenesis is largely unknown. Zimmers and colleagues investigated the effect of GDF-15 loss *in vivo* on hepatocellular carcinogenesis and found that genetic ablation of GDF-15 had no apparent effect on the HCC tumor formation rate, growth rate or invasiveness in a diethylnitrosamine-induced HCC mouse model.[37] However, our previous *in vitro* study demonstrated that GDF15 overexpression in Huh7.5.1 cells resulted in increased DNA synthesis, cell proliferation and enhanced invasiveness of the cells.[6] Therefore, elucidation of the biological function of increased GDF15 in liver disease pathogenesis will promote the potential application of GDF15 in diagnosis and targeted therapy.

In summary, we showed that GDF15 is a novel serum marker of HCC and LC and that its overall performance is satisfactory. However, some limitations need to be acknowledged. First, the specificity of GDF15 for HCC and LC needs to be better investigated to eliminate diagnostic interference by other diseases associated with GDF15 elevation, such as heart failure and especially other cancers. Second, larger cohorts should be considered in future studies to make more accurate associations. Even so, it appears promising to combine GDF15 with other biochemical markers and imaging methods to improve the diagnosis of HCC and cirrhosis. In addition, future studies should also be performed to clarify the mechanisms of cellular GDF15 production and release and to optimize and standardize quantitative assays with high reliability.

## Supporting Information

S1 Checklist STARD checklist. The STARD guideline check-list for diagnostic tests is provided in Supporting Information.
(DOC)
